# The Measurement of Domestic Abuse – Redeveloping the Crime Survey for England and Wales

**DOI:** 10.1007/s10896-023-00507-9

**Published:** 2023-02-15

**Authors:** Marianne Hester, Sarah-Jane Walker, Andy Myhill

**Affiliations:** 1 University of Bristol, Bristol, United Kingdom; 2 College of Policing, London, United Kingdom

**Keywords:** Domestic violence and abuse, Prevalence measure, Coercive control, Impact, Victim voice

## Abstract

**Purpose:**

In 2020 the England and Wales Office for National Statistics commissioned the research reported here to review the current questions on domestic abuse in the Crime Survey for England and Wales (CSEW) and to establish how better data for policy and practice might be produced. The CSEW is a representative population survey that since the early 2000s has provided ongoing measurement of domestic abuse via a dedicated domestic abuse module, with regular publication of headline prevalence and other descriptive data. At the same time the measurement of domestic violence in the CSEW has also been the subject of ongoing debate and critique, in particular whether it is appropriate to use catch-all prevalence measures in the context of policy, practice and commissioning of services.

**Method:**

The research included analysis of CSEW user survey data (N = 39), focus group and individual interviews with male and female victims/survivors (N = 11), consultation with core stakeholders (N = 18), and consideration of international surveys and recent legislation.

**Results:**

Current CSEW questions do not capture domestic abuse accurately or reflect lived experience, coercive control needs to be seen at the core of domestic abuse, and while physical assault is an important part of measuring domestic abuse establishing frequency through counting events is probably impossible.

**Conclusion:**

A fundamental rethink of the current CSEW self-completion module is required, with a wider set of questions about domestic abuse and impact. A revised module should identify and provide estimation of prevalence for different ‘abuse profiles’ that would complement improved headline measures and better inform policy and practice.

## Introduction

In 2020 the England and Wales Office for National Statistics commissioned a team of researchers at the University of Bristol (PI Hester) with colleagues from the College of Policing, Women’s Aid and the Men’s Advice Line to review the current questions on domestic abuse in the Crime Survey for England and Wales (CSEW) as part of work to redevelop the survey. The research took place between December 2020 and June 2021. This paper outlines the findings from the research, which involved a user survey, survivor and wider stakeholder consultation and mapping exercise and discusses the implications for the development of new domestic abuse questions for the CSEW.

The CSEW is a representative victimisation survey which asks people about their experiences of a range of crimes in the 12 months prior to the interview, as well as their attitudes towards different crime-related issues and perceptions of crime. It provides ongoing measurement of domestic abuse via a dedicated domestic abuse self-completion module The general survey is asked of around 34,500 people aged 16 and over resident in households in England and Wales every year. In contrast to police recorded crime, the survey also captures incidents that are not reported to the police and the estimates produced are unaffected by changes in police recording practice.

**Fig. 1:**
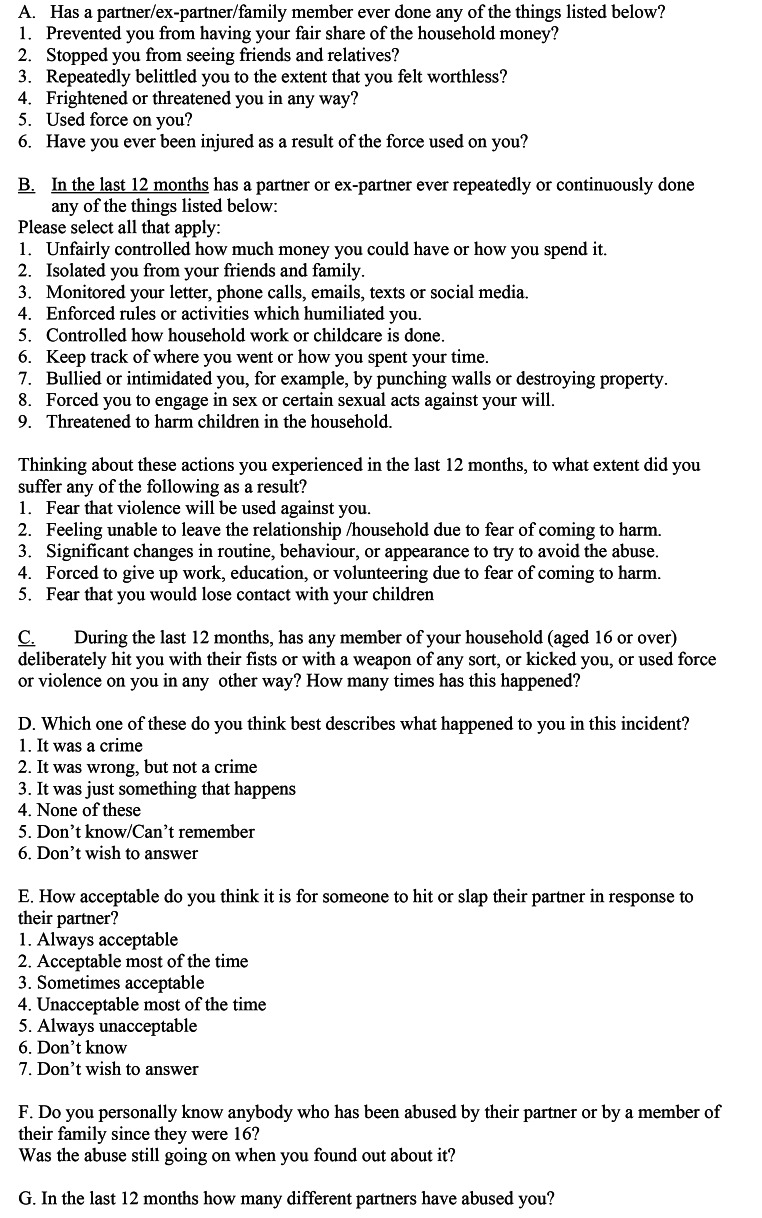
**– Questions on domestic abuse in the Crime Survey England and Wales and ONS trial questions tested in the study**

Self-completion modules were first introduced onto the CSEW in 1996 and have been included on a continuous basis since 2004. Introduced to collect information on topic areas that respondents could feel uncomfortable talking about to an interviewer, the modules have always included domestic abuse, sexual assault and stalking which at the time of the research were asked of all respondents aged 16 to 74. There are two relevant modules: the ‘Domestic abuse, sexual assault and stalking’ module and the ‘Nature of partner abuse’ module (see Fig. 1 sections A, C, D, E, F and G for the questions asked across the two modules that were tested in our study). Unlike many other surveys on domestic abuse, such as the European Union Agency for Fundamental Rights (FRA) survey (2014), the CSEW modules invite responses from both men and women. The general ‘main’ survey asks about one element of domestic abuse: incidents of violence (referred to as domestic violence), but the headline prevalence estimates for domestic abuse are produced from the self-completion module on ‘Domestic abuse, sexual assault and stalking’ as this provides a more complete and accurate measure of domestic abuse victimisation.

The measurement of domestic violence and abuse in sample surveys has evolved through several iterations (Walby & Myhill, 2001). Generic crime surveys were not designed to measure domestic violence specifically and were acknowledged to produce significant underestimates of prevalence. Surveys dedicated to measuring domestic violence, utilising the Conflict Tactics Scale (CTS, see Straus and Gelles, 1990) produced much higher estimates but were criticised for not situating acts of physical abuse or psychological aggression in context in order to ascertain meaning and impact (see Walby & Myhill 2001). A further development saw dedicated violence against women surveys developed in the US, Canada and other countries that sought to identify a wider context of non-physical coercion and control in addition to CTS-derived measures of physical violence and psychological aggression. These surveys produced still higher rates of prevalence (Walby & Myhill, 2001). Some national crime surveys, including the CSEW, borrowed from these state-of-the-art violence against women surveys to field revised question sets in dedicated modules within the survey, exemplified by the two self-completion modules in the CSEW.

While the CSEW self-completion modules exemplified the ‘state of the art’ regarding DA surveys at the time they were developed (Walby & Myhill, 2001), issues and gaps have been highlighted since then, and the measurement of domestic abuse has been the subject of ongoing debate. These debates have centred largely on how well headline prevalence data represents the lived experiences of survivors, and, in particular, whether it is appropriate to use catch-all prevalence measures in the context of policy, practice and commissioning of services. For instance, prevalence estimates from the CSEW suggest ‘one in three’ victims of domestic abuse are men but that proportion does not reflect the profile of victims that currently request support and intervention from support services and other agencies, as these are largely women (ONS, 2022). Men’s advocates suggest the barriers to reporting may be greater for men than for women, although women also face barriers to reporting (Myhill, 2017). While these remain as open and empirical questions, currently the balance of literature, homicide data and support sector opinion indicates that the abuse that requires intervention is gendered and predominantly affects women (ONS, 2022a; ONS, 2020; Women’s Aid, 2021). These are empirical issues that need to be addressed with a new approach in the CSEW.

In addition, there has in recent years been acknowledgement of coercive control as a key feature in domestic abuse, resulting in legislation in England and Wales in 2015 to criminalise controlling or coercive behaviour in an intimate or family relationship (Serious Crime Act 2015). The offence of Coercive Control specifies the repeated and continuous nature of the behaviours (which include a range of physical and non-physical behaviours) and the serious impact on the victim. The new Domestic Abuse Act 2021 (being debated in the UK Parliament when the research reported here began) extends the offence to cover post-separation abuse, introduces a new, statutory, definition of domestic abuse that includes economic abuse, and also introduces a specific offence of non-fatal strangulation. These are features that need to be incorporated (more robustly) in the CSEW question set.

We were therefore asked to consider how a new set of questions could be developed to accurately measure prevalence of domestic abuse including controlling or coercive behaviour; how to measure number of incidents or frequency of abuse; ongoing comparability of the data; and to take into account the limitations of the current module set up and space in the survey. In what follows we outline some of the issues highlighted from the wider literature and surveys regarding measurement of coercive control and frequency of abuse that informed our work, before moving on to outlining the methods and findings from the research.

### Considering Coercive Control

Coercive control has been arguably the most prominent concept in the national policy response to domestic abuse in recent years. Stark (2007, p.228) describes coercive control as “a pattern of behaviour which seeks to take away the victim’s liberty or freedom, to strip away their sense of self”. It is the impact of these behaviours which are particularly psychologically damaging to victims-survivors and it is these acts of undermining a persons’ sense of self, which keeps a victim within an abusive relationship irrespective of whether physical abuse is present or not. Herman (1992) makes connections between these tactics as used in domestic abuse and hostage contexts, recognising that both groups experience “terror, intermittent reward, isolation, and enforced dependency” (Herman, 1992: 83).

As Stark (2007) indicates, coercive controlling behaviour is particularly harmful to victims. Others concur, also indicating that coercive control can be seen as a measure of severity. Johnson (2008) identifies coercive controlling violence as the most severe and harmful form of domestic violence, and as an important distinction from ‘situational couple violence’. A study by Hardesty et al. (2015) to operationalise coercive control provides further evidence of the centrality of coercive control for identifying severity and differentiating between forms of intimate partner abuse. Analysis from the Fundamental Rights Agency (FRA) Europe-wide survey, which included the UK, also indicates the importance of measuring coercive control as a means of identifying especially serious domestic abuse resulting in high impact (Nevala, 2017). The FRA survey was only directed at women and shows how coercive controlling abuse differs from other forms of abuse against women as it involves more serious forms of violence and has a bigger impact in terms of its varied consequences. Nevala (2017) concludes that we need to differentiate between various types of intimate partner violence against women, including violence under coercive control, to ensure robust measurement. A survey of men in family doctor practices found that coercive controlling behaviour was also important to distinguish between the large number of men who reported some experience of situational and low coercively controlling behaviour and those who experience severe harmful domestic abuse (Hester et al., 2017). The authors found only one in forty men experienced such severe coercive controlling violence as victims specifically.

Stark suggests coercive control is specifically gendered and that “sexual inequality is the primary context for coercive control” (Stark, 2012, p206), although has also begun to consider experience of coercive controlling behaviours in same -sex relationships and by children (Stark & Hester, 2019) Myhill, (2015) in his analysis of CSEW data also found that women were more likely than men to experience ongoing abuse that resulted in negative consequences and agency intervention. Myhill (2015) concluded, however, that the existing CSEW questions were insufficient to capture the full range of behaviours and impacts associated with coercive control.

An initial attempt to measure coercive control in the context of the CSEW self-completion module was carried out by the ONS in 2017 (ONS, 2019). Half of the CSEW survey sample received a new set of questions with nine about coercive and controlling behaviour and five follow-up questions on impact (Fig. 1 questions B ). The other half of the sample received the three current non-physical abuse questions (Fig. 1 questions A1-3). The more tightly defined trial questions generated considerably lower estimates of domestic abuse. Overall, the trial questions were deemed largely unsuccessful, with further questions remaining regarding the difference in domestic abuse prevalence estimates generated by the two sets of questions, differences between men and women in the prevalence of domestic abuse, and whether the wording and other aspects of the questions may be drawing in people who are not victims, or be missing those who are (ONS, 2019, p2). These findings led to the commissioning of the research reported here.

### Measuring Frequency of Physical Violence

Not all commentators subscribe to coercive control as the primary framework through which to view and measure domestic abuse severity. Walby & Towers (2017) suggest the best way to assess severity and to foreground gender in the measurement of domestic abuse is to use existing crime codes and count the frequency of physical assaults. The authors argue that in domestic violent crime all physical violence can be conceptualised as coercive and controlling, and by looking at repetition and seriousness of violent offences this provides a better measure of domestic abuse prevalence and is a better way to foreground the gendered nature of abuse. However, other researchers have highlighted the limitations of focusing on physical violence which may be absent in the wider context of non-physical abuse (Myhill & Kelly, 2021; Donovan and Barnes 2021). Frequency of physical violence is none the less an important consideration and, as indicated earlier, also inextricably linked to the issue of measuring coercive control given that the underlying threat of physical violence is a core tactic present in many coercive and controlling relationships (Stark, 2007).

Moreover, there is also a wide consensus (though see Walby & Towers 2018) that not all abuse reported to population surveys will be continuous or coercive and controlling in nature and may not be recorded as violent offences (see Myhill 2017). Some may be one-off or isolated acts of physical violence, or psychological aggression. In addition, there is an emerging literature (see for example Ackerman 2016; Hamby, 2016) which suggests respondents report at least some proportion of non-abusive acts to population surveys such as the CSEW. This is likely due to the way ‘screener’ questions have historically been phrased deliberately to be free of a ‘crime context’ in order to encourage disclosure (see Walby & Myhill 2001). Thus experiences not necessarily deemed criminal offences, also need to be considered in the measuring of frequency.

### The Study

Based on the changing policy landscape and the issues and critical debates outlined above, our research aimed to review the existing and recently trialled questions on domestic abuse in the CSEW (see Fig. 1) to begin to establish how we might develop better data for policy and practice, especially as it relates to gender and lived experience. We were asked in particular to address how to measure prevalence of domestic abuse including controlling or coercive behaviour, and incidents or frequency of abuse. Moreover, replication and continuity have been an important feature of the CSEW and allowed the analysis of trends over time. Any changes to the CSEW may disrupt the existing time series and thus also had to be taken into consideration.

## Methods

Our methods involved analysis of recent CSEW user survey data (n = 39) looking at how academics, policy makers and others use data from the CSEW; focus group and individual interviews with male and female victims-survivors (n = 11 individuals); consultation with core stakeholders (n = 18); mapping of the current content and structure of the CSEW modules alongside user needs; and review of comparable surveys internationally to explore how they measure domestic abuse and what we might learn from them. Ethical approval was given by a University of Bristol Ethics Committee and by the Data Ethics Team at the UK Statistics Authority. Below we outline the methods in greater detail.

It should be noted that the research also involved extensive work to explore the survey modes that could usefully be employed in future iterations of the CSEW. However, we do not report on that aspect of the work here.

### Analysis of the ONS 2020 User Survey Data

The ONS carried out an open consultation in the form of a survey for key users of the CSEW data on domestic abuse in summer 2020, asking questions regarding their use of the data, whether continuation of the trend data (or ‘time series’) or improvement of the measurement of domestic abuse is preferable, and the relative preference of recording frequency versus the nature of abuses, amongst other aspects of how the CSEW is used. The survey had 14 tick-box and ranked questions, and space for free text responses. There were 39 responses, including from the charity sector, police and academia. The research team were provided with the survey data by the ONS and conducted descriptive statistical analysis on data from the 14 tick-box and ranked questions, and coded and analysed the free text responses thematically. We used our knowledge of the self-completion module, its content and how it is structured to feed into the coding and analysis of the survey data, paying particular attention to free text responses that provided context to the tick-box questions relating to ranking of priorities. Key themes were uses of the CSEW (including life-time prevalence, intimate partner and/or family abuse), improving domestic abuse measure versus time series, topic importance, and other issues.

### Focus Groups and Individual Interviews with Domestic Abuse Survivors

Consultation with domestic abuse survivors was carried out in late February-early March 2021, to gather survivor feedback on the existing domestic abuse questions. This was deemed a crucial aspect, drawing on survivor voices as ‘experts by experience’. We consulted 11 survivors of domestic abuse to obtain their views on the questions currently used within the CSEW. One online focus group had five female survivors, facilitated by Women’s Aid, and there were individual online interviews with five male survivors, facilitated by the Men’s Advice Line, and with one female survivor, facilitated by Women’s Aid. The survivors were from a range of age groups (18 years to late 60s), ethnicities (five participants identified as from Black and minoritised communities), had experience of domestic abuse from both hetero- and same sex partners (at least one participant identified as gay or bisexual) and at least one participant had a disability. The sample size and range of participants were agreed by the ONS to provide insight to varied populations and rich data, rather than representative data. Although it was a small sample, and thus had limitations, it provided useful explorative and indicative data with regard to the research topic.

The consultation presented the participants with the CSEW questions and the questions on coercive control tested by ONS in 2019 (as in Figure 1), in order to ascertain:

1. how well they think the current questions capture lived experiences of domestic abuse (partner / ex-partner and family abuse);
2. their views on the relative importance of some questions over others e.g. whether improving the measure of domestic abuse to capture coercive control is more or less important than counting the frequency of physical assault / continuing a comparable time series;
3. the potential impact (as a survey respondent) of being asked the current CSEW questions about the nature of domestic abuse.

The focus group and interviews were carried out online (via a secure version of Zoom). A Participant Information Sheet, Consent Form and additional information were provided to all potential participants ahead of the focus group and interviews. The additional information covered what the participants could expect on the day (structure / housekeeping / confidentiality/ rules of engagement etc.); a copy of the questions we wanted them to consider; and contact details for specialist domestic abuse support services. Particular attention was paid to confidentiality, given that these consultations were online and thus also required the participants – not only the researchers - to ensure all participants’ confidentiality, for example by making sure that the conversation could not be overheard by other adults. The researchers read the Consent Form aloud at the start of the focus group/ interviews, checking for understanding by participants and reminding them of their right to withdraw from the research. Three members of the research team participated in the focus group – one to facilitate, one to take notes, and one to manage the call (e.g. admitting anyone who joined after the start). Two researchers participated in each individual interview – one to facilitate and one to take notes. Back-up recordings were made (with participants’ permission) to act as aide memoire for finalising the notes and the audio recording only stored directly on the university’s secure server. The feedback gathered during the focus group and interviews was coded thematically to link to and build on the research questions and issues identified in the wider literature and recent legislation. These key themes were: headline domestic abuse measure, measuring coercive control, measuring the impact of domestic abuse, measuring the frequency of physical abuse, and attitudes to and perception regarding domestic abuse.

### Consultation with Core Stakeholder Group

Following the analysis of the user survey data and consultation with survivors (focus group and interviews), a core group of stakeholders were sampled purposively to provide a mix of policy makers, NGOs and academics, and consulted in February 2021 to review and challenge emerging findings about the current headline measure of domestic abuse, measuring the frequency of acts of abuse, measuring coercive and controlling behaviour, and measuring impact. The core group were consulted again in June 2021 regarding the results and recommendation from the research. Participants in the group included representatives from the Domestic Abuse Commissioner’s Office, ONS; Home Office; Kantar Public (who administrate the CSEW); universities; College of Policing; and key survivor support NGOs (Women’s Aid Federation England; IMKAAN and Respect). The consultation took place online (via Zoom) and lasted 90 min. A recording was made (with participants’ permission) and was used to aid in the writing up of meeting notes.

### Mapping Exercise

The current content and structure of the CSEW modules was mapped in order to build a means of comparing existing content with user needs (from the user survey) stakeholder input (consultations with survivors and other key stakeholders – see below), plus any issues from the wider literature and issues related to the Domestic Abuse Act 2021 at that time being discussed by Parliament. As part of the mapping exercise, the content and structure of other international partner abuse surveys were also identified for comparison. Material from the mapping exercise was used in formulating the recommendations.

## Findings

Below we discuss the findings in relation to the different data sources.

### 2020 CSEW User Survey

Analysis of the 2020 ONS online survey for users (n = 39) regarding use of domestic abuse-related data in the CSEW looked at questions regarding use of the data, whether continuation of the time series or improvement of the measurement of domestic violence is preferable, and the relative preference of recording frequency versus the nature of abuse. Respondents stated they use the survey for the following purposes (respondents could choose more than one; 20 respondents selected only one purpose, 18 selected more than one):

- 56 (n = 22): Improving policy or processes.
- 51% (n = 20): Planning service provision.
- 36% (n = 14): Academic research.
- 31% (n = 12): Other, including (not all listed here as some repetition or falling under above categories): awareness raising and campaigning work; non-academic research; government research; to evidence need e.g. in training sessions; funding applications; understanding current trends (including to help develop policy); comparing against recorded crime figures.

A majority (57%, n = 20) of the 35 respondents who answered whether the measure of abuse should be improved rather than continuing with a comparable time series, stated that improving the measure of domestic abuse is more important. Among respondents who use CSEW data for academic research and/or for planning service provision (and in some cases also for other purposes), significantly more favoured improving the measure over continuing with a comparable time series (n = 9 vs. n = 2 among those who use it for academic research, n = 10 vs. n = 3 among those who use it for service provision). For respondents who use CSEW data for improving policy processes (and in some cases also for other purposes), there was very little difference (n = 8 vs. n = 7, with n = 5 not having a preference).

Respondents expressed substantial support for continuing to ask about experiences of domestic abuse from the age of 16 (‘lifetime’ prevalence). Those who answered that they supported this approach, felt strongly about the importance of this measure in showing domestic abuse experiences across the life course and the prevalence of domestic abuse in society, and using this information in policy planning and service provision.

Respondents overwhelmingly (95%, n = 37) favoured producing separate estimates of domestic abuse by a family member versus domestic abuse by a partner/ex-partner. One respondent elaborated:

Both are needed. Partner / ex-partner abuse and family abuse have different drivers and therefore need separate policy responses. It is crucial that we are able to measure both going forward.

Approximately three-quarters of respondents (74%, n = 29) stated they use data from the three-yearly nature of partner abuse module. Within the module topics, there was relatively little variation in terms of which topics respondents reported using, although ‘Physical/emotional impact of the abuse’ stands out as being used by 93% (n = 27) of these respondents. Some respondents felt the interlocking nature of types of domestic abuse means the linking of data across the survey and/or to specific perpetrators is important. ‘Ideally we would have more information between the victim and the perpetrator from the CSEW’. Respondents also made specific suggestions for additional topics to be covered, including technology facilitated abuse.

### Survivor Feedback on CSEW Content and Structure

The feedback from survivors in the interviews and focus groups regarding the current questions in the CSEW, suggested a strong desire and need for improving the questions on domestic abuse in terms of both content and structure. A number of key themes were identified, detailed further below:

1. Augmenting and future-proofing the questions (content).
2. The separation of non-physical coercions and control from physical assault (content & structure).
3. Reflecting the subtleties of coercive control (content).
4. The importance of impact (content & structure).
5. Measuring the frequency of physical assault (content).
6. Question areas for possible removal (content).

#### Augmenting and Future-proofing the Questions (Augmenting Behaviours, Improving Wording, Reducing Potential Bias)

In terms of content, feedback from the survivors suggested that the current questions (both the original questions and the more recent trial questions on coercive control and impact – see Fig. 1) would not necessarily capture all the abusive and controlling behaviours they had experienced themselves. They suggested the current questions would not go far enough to reflect the lived experience of domestic abuse amongst all groups (including Black and minoritised and/or disabled women, and men) and would need amending and expanding to better capture – and thus improve – the headline measure of domestic abuse. Feedback also suggested that the existing measure needs updating to fit the present context (e.g. operation of finances and methods of communication have changed in the last 20 years). These issues related mostly to the female participants, while the men interviewed tended to agree that the list of behaviours (both original and more recent trial questions) were ‘relatively comprehensive’ and mostly reflective of their experiences. However, one male survivor did suggest that some of the examples of behaviours listed in the current and trial questions (such as ‘punching walls’) were biased towards female victims/male perpetrators.

#### Abusive Behaviours not Reflected in the Current Questions

Survivors, especially the women, felt several examples of abusive behaviours were missing from the current questions, which might stop people proceeding with the survey if they feel it does not cover their own experience. They suggested a myriad of abusive behaviours that would better reflect lived experience of domestic abuse and help future survey respondents better identify with the examples given. These included (mainly from female survivors):

- Gaslighting (making the victim question themselves, their sanity, their thoughts and decisions).
- Use and abuse of pets and/or children.
- Being threatened around immigration status.
- Strangulation and choking.
- Withholding essential care (e.g., food / medication / health care / disability aids).
- Threatening to harm or kill themselves if survivor leaves.
- Threatening survivor with the authorities / trying to use the system against them.
- Making threats against survivor’s family members.
- ‘Vicious cycle’ of love-bombing and withholding affection.

Both male and female survivors suggested that the following were also missing:

- Purposely disturbing the survivor’s sleep / routine.
- Threatening to stop survivor having contact with their children.

The participants deliberated whether it would ever be possible have a comprehensive list of behaviours that would potentially reflect everyone’s experience of domestic abuse.

#### Updating the Wording of Current Questions

Wording related to economic abuse and surveillance were specifically deemed to require updating. Both female and male participants suggested that how day-to-day finances operate has changed over the years and thus the wording of questions such as preventing access to a ‘fair share of the household money’ might need to be revised to be more specific because what is fair to one person may not be fair to another. It was also suggested the concept of economic abuse goes beyond controlling the ‘fair share of money’ and needs to incorporate other methods of financial manipulation by the abuser. Survivors described being persuaded to take on debts in their name (e.g. mortgage and credit cards); being prevented from living within their means; having access to their credit card or benefits controlled (e.g. by taking away card and/or PIN); being made to feel financially unstable or threatened with the household budget being ‘bled dry’.

In relation to the existing question on surveillance (‘monitored your letter, phone calls, emails, texts or social media’) it was highlighted that primary methods of communication have changed since the survey began thus it might be better to change the order of wording so that greater emphasis is placed on social media as the main method of communication rather than letters.

#### Reflecting Subtleties of Coercive Control

A key theme from the survivor feedback was the difficulty of reflecting the subtle, insidious nature of coercive control. Participants suggested that any questions need to reflect that that coercive control is subtle, manipulative, insidious, and not always overt. The female participants in particular suggested many survey respondents may not recognise the coercive control or abuse against them as abuse while they are still in that relationship and would not have identified with the examples given in the current questions,

*All of those* [abusive behaviours] *applied to me. But, before I had fled, I wouldn’t have recognised any of those at all…I think that’s the problem with coercive control. When you’re in it you don’t actually recognise it. I mean you might get angry, you might get upset. But you don’t actually realise what’s going on (female survivor – FG1)*

Reflecting on the question about controlling how the housework or childcare is done, one male survivor suggested that individuals have their own preference for how tasks are done and warned that many households might agree with this statement, but it may not necessarily equate to abuse.

*More a case of inspecting and monitoring your work to see if it meets perpetrator’s expectations, with resulting fear in victim that they’re not doing it right and fearing negative consequences if you’re not doing it the way the perpetrator wants. Or being threatened with harm if you’re doing it wrong (male survivor -MV5)*.

Another, female, survivor stated that she did not see it as controlling or abusive when her partner was making her do all the housework or not letting her go in the garden etc. Her husband would also prevent her from going out by picking fights with her when she was due to go out, but at the time she did not see it as him preventing her from going out,

*He was stopping me doing it without me realising it (female survivor- FG2)*

One participant recalled how, at the time, she mistook her partner’s constant surveillance of her movements as an act of care rather than seeing it as controlling behaviour. Consequently, participants proposed any questions designed to capture domestic abuse need to be very carefully crafted to help respondents recognise abusive behaviours as applying to them if they are still in the relationship.

Participants provided numerous examples of how manipulation by perpetrators worked in reality. One participant reported that while her ex-partner did not directly prevent her having what may be considered a ‘fair share of the money’, he did tell her ‘*how she could spend it’ (female survivor – FG3).* Another suggested that a victim may not even perceive it as ‘unfair’ while in that relationship, ‘*You might think ‘Oh no, no, no he lets me have my money’ (female survivor - FG2)’.* Others explained that while their partner did not directly prevent them from seeing friends or family, they would put up barriers or make it difficult to do so, for example, by either constantly ‘badmouthing’ family /friends, or making them feel guilty about seeing family/friends, or regularly interrogating communication with them to the point where they then would feel anxious about seeing or talking to particular people. Survivors described it as putting so much energy into the relationship that they would end up withdrawing themselves from their social circle or being made to feel uncomfortable about contacting friends or relatives,

*I couldn’t say I was stopped from seeing friends. I stopped myself, based on how I felt, because of the fear around it. (male survivor – MV5)*

A challenge identified was that the situation is often ‘not as black and white’ as the current questions/examples in the CSEW and trial questions would suggest. Survivors described being made to feel guilty or bad for doing certain things, which would discourage or stop them from doing those things, but they would not have recognised- and thus not reported- that this was because their partner had directly prevented them from doing so.

*It is still abuse/coercive control even if partner does not overtly prevent you from doing things. An awful lot of them don’t prevent you, they just manipulate you all the time (female survivor-FG1)*

Similarly, on being asked whether you had been ‘forced to give up work, education, or volunteering due to fear of coming to harm’, survivors reflected that a victim may not have been directly ‘forced’ to give it up, but may have been made to feel guilty for being in work or education where a perpetrators might use language such as ‘It would be better for all of us if you…[left work etc.]’ So that behaviour and the impact can be a lot more subtle,

*They make you feel uncomfortable about it, end up manipulating you to give things up, change your behaviour. And you want to please. So it’s more about how it makes you feel. (female survivor -FG5)*

#### The Importance of Measuring Impact

Reflecting on the current survey and trial questions used to capture impact participants also suggested there were a number of important impacts missing, including:

- Fear of losing your home/becoming homeless (e.g. if the house is in perpetrator’s name and especially if you have children).
- Fearing for your immigration status.
- Fear that leaving the relationship will result in coming to harm.
- Fear that you will not be able to manage on your own, because the perpetrator has undermined you so much (lack of confidence rather than lack of money).
- Perpetrator refusing to leave the family home (e.g. if house is in survivor’s name).

There were also comments regarding specific words and terminology. For instance, with regard to the wording of the question ‘repeatedly belittled you to the extent that you felt worthless’ female participants suggested that feeling ‘worthless’ could be quite far down the line, but it is still abuse even if you don’t feel completely worthless yet. Coercive control is more about being made to question your self-confidence and a survivor might not feel ‘worthless’, but they may still have been made to doubt themselves / their self-confidence. As well as raising issues around terminology this relates back to the importance of recognising the impact of abuse on the victim rather than asking about specific behaviours perpetrated against them.

Reflecting on the response choice of whether the impact suffered was ‘a little’ or ‘a lot’, survivors reported that ‘fear that violence will be used against you’ has a significant impact. Having these specific options could have the effect of belittling that feeling of fear. It was also unclear to some the difference between the response options ‘very much’ and ‘quite a lot’ previously tested by ONS (ONS, 2019) suggesting this would again be difficult to answer. Another pointed out that the level of abuse can fluctuate, and thus is not the same all of the time.

#### Asking About Physical Assault Separately from Non-physical Coercion and Control

Given the definition of Coercive Control in the recent legislation on Domestic Abuse (which includes wide range of behaviours) participants felt confused about why questions on physical violence were asked separately from the questions on coercive control,

*Is physical violence not included here? Is this meant to capture CC without violence? CC behaviour where there is also violence seems to be more damaging to an individual’s mental health (male survivor- MV3)*

*Even though the crime survey is trying to put stuff about CC in there, it still seems more biased towards physical violence. (male survivor-MV2)*

#### Measuring the Frequency of Physical Assault

Survivors were in agreement that they would find it difficult, if not impossible, to count the number of times they had been physically assaulted in the past year. They suggested physical assaults could often be sporadic and survivors also tend to ‘block it out’:

*I think about it now and I still can’t remember. (female survivor – FG2)*

*People would find it hard to count and so I would question how honest or accurate responses to this would be (male survivor - MV4)*

*As a victim you begin to grade the assaults. And so naturally you start to remember the more significant ones (male survivor – MV3)*

A number of survivors suggested using grouped responses e.g. 1–5 times, 6–10 times etc. or perhaps giving the option of responding ‘more than once in the past year’, ‘every 3 months’ or ‘every 6 months’ etc.

#### Question Areas for Possible Removal

Within the current CSEW survey there are questions regarding whether what respondents experienced was a crime; about their attitudes to domestic abuse; and whether they know anybody else that has been abused and what they did about it. Survivors generally agreed these questions could be considered for removal if space was required in the survey.

### Core Stakeholder Group Feedback

#### The Current Headline Measure of Domestic Abuse

It was put to the participants that the current headline measure of domestic abuse represents an established trend/time series, that the measure has been subject to criticism for de-gendering domestic abuse and not representing adequately the lived experiences of survivors, and that the trend has in any case been interrupted due to the impact of the pandemic on fieldwork for the survey. Most participants, even if expressing a desire to continue the current time series, acknowledged that the current headline measure risks de-gendering domestic abuse, that the experience of abuse can differ between different respondents to the survey, including in some cases men and women, and that current questions on abusive behaviours are not detailed enough. Most participants, particularly specialist support services, while recognising the importance of time series data, preferred an improvement in the headline measure.

Participants raised various reasons why it is more important to *improve the headline measure* than preserve the current time series, including:

- The current headline measure is not capturing prevalence adequately because it does not reflect accurately who is experiencing what from whom, or the impact or context of the abuse and these are important to capturing the gendered nature of domestic abuse.
- The measure should be updated to reflect changes in legislation, including the Domestic Abuse Act 2021 and the offence of controlling and coercive behaviour (Serious Crime Act 2015).
- The current headline measure does not cover all forms of domestic abuse, including so-called ‘Honour-based violence’.

Participants did however also suggest advantages to *preserving the current time series/measure*:

- The CSEW is the main source of prevalence data on domestic abuse.
- Due to the Covid-19 pandemic in particular, reinstating the current time series (and current measure) would permit assessment of the potential impact of the pandemic and lockdowns on domestic abuse prevalence (although in reality the interruption of the time series due to Covid-10 mitigates against this).

#### Measuring Frequency of Acts of Violence

It was put to participants that asking victim-respondents how many times they have suffered specific acts of abuse is one possible way of reflecting gender differences in abuse (Walby & Towers, 2017). The number of assaults suffered in the past 12 months is measured in the main CSEW question set which asks about a range of different crimes, but not the self-completion module on domestic abuse. It was pointed out that asking the frequency of abusive acts in the self-completion module may add significantly to the length of the module. Generally, participants saw frequency as an important measure, but also raised concerns about how to ask about frequency, the relative importance of different forms of abuse, and how to capture patterns of abuse. It was suggested it can be difficult for victims/survivors to put a number to incidents, so perhaps using grouped responses might be better, e.g. asking if they suffered abuse ‘once a week’ or ‘more than once a week’. In this vein, there were concerns regarding the use of incident analysis, and how accurate such measures may be considering the difficulty of many survivors to recall numbers of incidents accurately. This raised the question: what is more important: a victim experiencing e.g. five acts of physical abuse or another victim experiencing one act of rape? From this stakeholder’s point of view, the question seems to be - what matters to the individual victims-survivors, or what matters from a policy point of view? Who is this survey ultimately for? It was argued that it would be important to ask about frequency of abuse, because there are multiple users of data from CSEW. For instance, the police and accident and emergency departments often deal in episodes and need a measure of episodes and thus need a measure of frequency. 

While there was consensus that capturing frequency of abuse is important, the issue was raised about the inability of the current CSEW data to distinguish between those survivors who are experiencing a sustained pattern of abuse over time, from those individuals who have experienced a single incidence of abuse, or if incidents may be included which are an act of retaliation in a context of a sustained pattern of abuse against them. This is a challenge and concern within the domestic abuse sector regarding the current use of domestic abuse statistics, in terms of truly capturing what we mean when we define domestic abuse more generally.

#### Measuring Coercive and Controlling Behaviour

It was put to the group that controlling and coercive behaviour has been a criminal offence in England and Wales since 2015, and that asking respondents about non-physical coercion and controlling behaviour in addition to physical violence may better reflect the lived experiences of some victims-survivors. It is also a possible way of reflecting gender differences in abuse as police data suggests that perpetrators of this offence are overwhelmingly men.

Generally, respondents considered coercive control as a core feature of domestic abuse, and that this may include non-physical coercion and physical violence. But they saw this as creating difficulties and complexities for measurement. Respondents suggested that the impact of the behaviour would need to be asked about, the variety of coercive and controlling behaviours and different perpetrators involved would need to be included, and some victims may not recognise coercive control. Respondents / stakeholders agreed that non-physical coercion and control and physical abuse are very inter-linked, and the definition of coercive control includes that pattern of continued / repeated abuse and its impact. It is difficult therefore to unpick or separate them. While it was suggested that male survivors do not yet have the language about coercive control to be able to express it in those terms it was agreed that “*measuring the impact of abuse is part of measuring coercive control - which cannot be done using the existing questions… If we get to the heart of coercive control, then we get to the heart of who is experiencing domestic abuse” (specialist service).*

It was queried whether there would be a breakdown of coercive control and a way of differentiating between the different behaviours. For example, the domestic abuse questions need to be able to pick up on immigration abuse or honour mediated abuse which can be perpetrated not only by an intimate partner but also by other family members and involve coercive control. Frequency and impact are vitally linked within that.

#### Measuring the Impact of Domestic Abuse

It was outlined to participants that the impact of abuse from intimate partners is currently measured (once every three years) in the ‘nature of partner abuse’ module through questions on physical injury. Participants indicated that a range of physical and non-physical impacts would more accurately reflect service user experiences, with one stating a preference for the use of the term ‘harm’ as opposed to ‘injury’ (which only resonates with physical violence), with another raising the importance of different measures for physical and psychological harm as they are essential for commissioning (agreeing to pay for) services. Another suggested it is important to measure the immediate and longer-term impact on survivors and that support services need a solid evidence base.

A number of respondents suggested there needs to be more detailed demographic data so that factors such as ethnicity, age and children can also be taken into account. It is important to have this data in order to be able to begin to address structural inequalities and how they intersect with domestic abuse. Another felt it is important to measure impact as a way of distinguishing who is really experiencing domestic abuse, and who might be a primary perpetrator reporting their experience of retaliation violence or someone who is in an unhappy relationship rather than an abusive one. It was also suggested that questions and data relating to impact of abuse and the physical and emotional harms would be valuable e.g. when calculating the economic cost of domestic abuse.

## Discussion

### Headline Measure of Domestic Abuse

Overall, the feedback suggests that improving the headline measure is more important than continuing the current time series (if it was a question of either/or). The majority of people consulted, both core stakeholders and survivors, felt that the CSEW’s headline measure of domestic abuse needs to be improved to ensure it reflects a more accurate picture of domestic abuse prevalence and experience, including its gendering. Though not accepted by all in the field, we believe the balance of opinion and research – including studies of the gender pay gap, and women’s disproportionate share of household work and caring responsibilities – supports the privileged position of men in society and that this structural inequality is an important context when considering domestic abuse. Moreover, our own and the work of others has highlighted the potentially different range of behaviours experienced by survivors of domestic abuse subject to different intersecting inequalities and circumstances (Donovan & Hester, 2017; Gill & Walker 2020). We are mindful that any development of survey questions needs to be relevant for different groups of survivors, while focusing largely on common elements relevant to all gender and other identities. It should be noted that there was a large degree of consensus among our small, but diverse, sample of survivors regarding many of the areas addressed in the research.

If the coercive control questions recently tested by the ONS (ONS, 2019), involving intent of the perpetrator, pattern of behaviour, and impacts of coercive control, were included in the CSEW the survey would cover a wider range of domestic abuse related topics than other international domestic abuse surveys such those in the US (Smith et al., 2017), Canada (CGSSV, online) and New Zealand (NZCSS, 2021). However, comparison with the content of other domestic abuse surveys also shows that some, such as the European Union Agency for Fundamental Rights (FRA) survey (2014), the Australian Longitudinal Survey of Women’s Health (ALSWH, 2019) or the National Intimate Partner and Sexual Victimisation Survey (US) (Smith et al., 2017), contain much more detailed items within their headline measure of domestic abuse.

The research identified a number of limitations regarding the current domestic abuse questions, which are however mentioned in other surveys. Various topics should now be considered for inclusion to ensure an up-to-date and robust approach, such as technology- facilitated abuse, withholding essential care and threat to harm self. Online and digital forms of coercive control include the monitoring of social media profiles or emails, abuse via Facebook or Twitter and using GP locators (Harris & Woodlock, 2018). Other domestic abuse surveys include questions on this e.g. Australian Personal Safety Survey (PSS, 2017) asks whether ex/partner *‘Controlled or tried to control where they went or who they saw (e.g. constant phone calls, GPS tracking, monitoring through social media websites)’.*

Research suggests that women with disabilities may suffer multiple forms of abuse, including disability related abuse and neglect such as withholding medications, denying access to mobility devices, neglecting personal care, and preventing attendance at doctor’s appointments and that abuse can also be contextual (Plummer & Findley, 2011; Thiara et al., 2011). The Australian Personal Safety Survey (PSS, 2017) includes a question on withholding of essential care: *‘Deprived them of basic needs such as food, shelter, sleep or assistive aids’*).

Threatening to harm or kill themselves if the survivor leaves is addressed in a number of other domestic abuse surveys e.g. Scottish Crime and Justice Survey (SCJS, 2019) includes the item *‘Threatened to kill or attempted to kill themselves as a way of making you do something or stopping you from doing something’*.

Also, the current questions do not capture domestic abuse accurately. It does not necessarily reflect lived experience of domestic abuse, especially amongst Black and minoritised communities and people with disabilities. For example, the domestic abuse question needs to be able to pick up on immigration abuse or honour mediated abuse which can be perpetrated not only by an intimate partner but also by other family members. This reflects research which suggests any new definition of domestic abuse must recognise the specific dynamics and needs of migrant women, that both intimate partner and non-intimate partner abuse may involve ‘honour’, and that abuse from intimate partners and family members may be labelled ‘honour-based violence’ when they relate to minority ethnic or migrant victims and perpetrators but are actually wider concerns (Bates, 2020; Siddiqui, 2014).

In whatever way the survey is structured, we suggest it will always be possible to generate a ‘global’ or headline measure of prevalence: the proportion of respondents who have experienced one or more acts of abuse either since age 16, or in the past 12 months. We believe it is important therefore that these headline measures are both accurate and inclusive. Development work will be required to revise headline measures, and the nature of these measures may be influenced by decisions around whether and how to measure coercive control, in particular. The current time series can be preserved while this development work takes place, and different measures could be run concurrently (split sample), or in alternate survey sweeps initially. In the longer term, however, we believe it is not desirable to have multiple headline measures, and in any case the time series has been disrupted by the Covid-19 pandemic. We question ultimately the value of preserving trends for measures that a majority of stakeholders agree are not fit for purpose. Having multiple headline measures would also do little to resolve current debates around prevalence and would lead to different stakeholders using the measure that most suited their purpose.

### Measuring Coercive Control

Both survivors and other core stakeholders in our research recognised that coercive control is at the core of domestic abuse and cannot be separated from other abusive behaviours, including the more serious and harmful forms of physical violence that often underpins coercive control (see also Nevala 2017). Several CSEW users noted that data on the scale, impact and nature of abuse are equally important for understanding domestic abuse as they are interlinked.

A key issue raised, however, was the difficulty of reflecting the subtle and insidious nature of coercively controlling behaviours and the possibility that many victims will not necessarily recognise such behaviours whilst still in the relationship. Thus, while a respondent may be experiencing some or all of the abusive behaviours listed in the survey questions, they may not answer affirmatively if they are still in an abusive relationship. When discussing their lived experiences of domestic abuse in response to whether the current survey questions would have reflected /picked up their experience, survivors suggested it is easier to recognise how the abuse / abuser made them feel or behave. This would suggest that questions regarding the impact of abuse on the victim-survivor (e.g. levels of fear or changes in their own behaviour as a response to the manipulation of the abuser) may mean a respondent is more or less likely to recognise and thus respond to/ answer affirmatively to these questions. This may also show up differences in impact of abuse between women and men who tend to report different social consequences of intimate partner violence (see Nybergh et al., 2013).

We acknowledge that significant issues remain relating to measuring coercive control, and that there is no agreed measurement instrument internationally. We think there needs to be significant development and testing undertaken to determine whether it is possible to measure coercive control robustly in the context of the CSEW. This development work should involve in-depth cognitive testing with both male and female survivors, as well as respondents from a general population sample. Issues with measuring coercive control include:

- Representing adequately the wide range of abusive behaviours in a relatively small question set.
- Do we keep physical violence questions separate from questions on non-physical coercion and control?
- Ensuring that behaviours reported to the survey are at a threshold that can be considered abusive.
- Designing questions that are appropriate for and reflect accurately the experiences of both female and male victims.

### Measurement of Impact

While the list of abusive behaviours in the current survey question(s) act as a ‘prompt’ to experience, measuring the *impact* of domestic abuse experienced is vital for assessing the harm and severity of abuse. Research shows the importance of taking into account impact alongside experience of abuse if we are to understand the nature of abuse for women and men and the implications for policy and practice (Hester et al., 2017). When discussing the current questions, the impact of the abuse on the survivor participants was often at the forefront of their discussions and used to frame their experiences. Coercive controlling violence requires particular attention to the safety of victims, including understanding of the effects of living in an on-going context of fear (Stark, 2007; Williamson, 2010).

The feedback from survivors supports an emphasis on impact as a more appropriate and reliable measure of domestic abuse rather than the behaviours experienced. This would also reflect the law on coercive control, where impact has to be demonstrated to record a crime. Currently the CSEW survey foregrounds act-based questions. Stakeholders and survivors emphasised the importance of measuring impact as a means of ascertaining severity and harm of domestic abuse. The work of Myhill (2015) and Hester an colleagues (Hester et al., 2010) involving CSEW and other survey questions highlight the importance of analysing impact alongside forms of abuse as a means of identifying different levels of harm and coercive control. Analysis conducted for a previous study (Hester et al. 2010) established thresholds across abuse and impact scales to ascertain severity of reported experiences which allowed a distinction between those experiencing behaviours with relatively little impact and those experiencing more severe impacts from coercive and controlling behaviours (Hester et al., 2010; Hester et al., 2017). We suggest that such an approach needs to be considered to assess severity profiles. This also raises the crucial issue whether the survey should prioritise capturing the impact of abuse on respondents ahead of trying to capture domestic abuse through act-based questions, as the latter may never be exhaustive enough to capture all experiences.? We believe that measuring impact is essential, that it should be measured in relation to global prevalence and propose that further work should be developed in this area.

### Measurement of Physical Assault Should be Disaggregated

In recent years, physical assault has been measured in the self-completion module by an aggregated screener question comprising acts ranging from pushing and slapping to choking and use of weapons. Based on our study aims we believe it is essential for identifying different abuse profiles for levels of physical assault to be disaggregated. In particular, strangulation/choking is a highly coercive form of sub-lethal violence, and non-fatal strangulation has now been made a criminal offence (Domestic Abuse Act, 2021). As well as the frequency of violence, the severity of violence is also a key means of discriminating abuse likely to come to the attention of support services and statutory agencies.

### Further Consideration Should be Given to Measuring Frequency of Physical (and Sexual) Victimisation in the Self-completion Module

The work of Walby & Towers (2017) has demonstrated that measuring the frequency of physical assaults is a helpful and revealing way to identify different profiles of abuse. It is clear, however, that there are also considerable challenges to measuring the frequency of acts of abuse, in the context of the self-completion module. Survivors who participated in our research had reservations about measuring frequency, both in terms of recall and appropriateness, but further development work could determine a viable approach.

We also believe measuring the wider context of ongoing, non-physical abuse as well as the frequency of physical violence is crucial to differentiating different profiles of domestic abuse to inform policy and practice. The absence of context makes many of the existing follow-up questions in the current CSEW nature of partner abuse module – reporting to the police and seeking help from specialist support services, for example – impossible to interpret sensibly.

## Conclusion

The Covid 19 pandemic, which has made the application of the CSEW as a face-to-face survey more difficult, has provided a good moment in time to rethink the content and approach. A revised module could identify and provide estimation of prevalence for different ‘abuse profiles’ that would complement improved headline measures and better inform policy and practice. In particular, it should be possible to differentiate victims of ‘one-off’ or infrequent abuse that has little or no impact from those suffering ongoing, coercive abuse (Hester et al., 2010; 2017). And within the latter group, it could be possible to identify victims for whom the abuse is mainly repeated physical assaults, victims who suffer primarily non-physical coercion, those who experience both physical and non-physical abuse, those who also experience sexual victimisation and post-separation stalking, and so on. Identifying different abuse profiles will allow us to see if different victim profiles will have different characteristics in terms of sex or gender, the impact of abuse, reporting and help-seeking, all of which are important for policy and practice. The research will thus enable a new comprehensive approach to domestic abuse surveys to be developed, that is rooted in survivor experience and linked to user needs.
